# Comparative transcriptional profile of the fish parasite *Cryptocaryon irritans*

**DOI:** 10.1186/s13071-016-1919-1

**Published:** 2016-12-07

**Authors:** Ze-Quan Mo, Yan-Wei Li, Hai-Qing Wang, Jiu-Le Wang, Lu-Yun Ni, Man Yang, Guo-Feng Lao, Xiao-Chun Luo, An-Xing Li, Xue-Ming Dan

**Affiliations:** 1 College of Marine Sciences, South China Agricultural University, Guangzhou, 510642 Guangdong Province People’s Republic of China; 2 School of Bioscience and Biotechnology, South China University of Technology, Guangzhou, 510006 Guangdong Province People’s Republic of China; 3 State Key Laboratory of Biocontrol, School of Life Sciences, Sun Yat-sen University, Guangzhou, 510275 Guangdong Province People’s Republic of China

**Keywords:** *Cryptocaryon irritans*, Fish parasite, Transcriptome

## Abstract

**Background:**

*Cryptocaryon irritans* is an obligate ectoparasitic ciliate pathogen of marine fishes. It can infect most marine teleosts and cause heavy economic losses in aquaculture. There is currently no effective method of controlling this disease, and little information is available regarding the genes involved in its development and virulence. We aimed to investigate the distinct features of the three major life-cycle stages of *C. irritans* in terms of gene transcription level, and identify candidate vaccines/drug targets. We established a reference transcriptome of *C. irritans* by RNA-seq.

**Methods:**

Three cDNA libraries using total poly(A)^+^ mRNA isolated from trophonts, tomonts, and theronts was constructed and sequenced, respectively. Clean reads from the three stages were *de novo* assembled to generated unigene. Annotation of unigenes and transcriptomic comparison of three stages was performed.

**Results:**

Totals of 73.15, 62.23, and 109.57 million clean reads were generated from trophont, tomont, and theront libraries, respectively. After *de novo* assembly, 49,104 unigenes were obtained, including 9,253 unigenes with significant similarities to proteins from other ciliates. Transcriptomic comparisons revealed that 2,470 genes were differentially expressed among the three stages, including 2,011, 1,404, and 1,797 genes that were significantly differentially expressed in tomont/theront, tomont/trophont, and theront/trophont pairwise comparisons, respectively. Based on the results of hierarchical clustering, all differentially expressed genes (DEGs) were located in five major clusters. DEGs in clusters 1 and 2 were more highly expressed in tomonts than in other stages, DEGs in cluster 3 were dominant in the tomont and trophont stages, whereas clusters 4 and 5 included genes upregulated in the theront stage. In addition, Immobilization antigens (I-antigens) and proteases have long been considered major targets for vaccine development and potential drug targets in parasites, respectively. In the present study, nine putative I-antigens transcripts and 161 protease transcripts were found in the transcriptome of *C. irritans*.

**Conclusion:**

It was concluded that DEGs enriched in tomonts were involved in cell division, to increase the number of theronts and ensure parasite continuity. DEGs enriched in theronts were associated with response to stimuli, whereas genes enriched in trophonts were related to nutrient accumulation and cell growth. In addition, the I-antigen and protease transcripts in our transcriptome could contribute to the development of vaccines or targeted drugs. Together, the results of the present study provide novel insights into the physiological processes of a marine parasitic ciliate.

**Electronic supplementary material:**

The online version of this article (doi:10.1186/s13071-016-1919-1) contains supplementary material, which is available to authorized users.

## Background

**Fig. 1:**
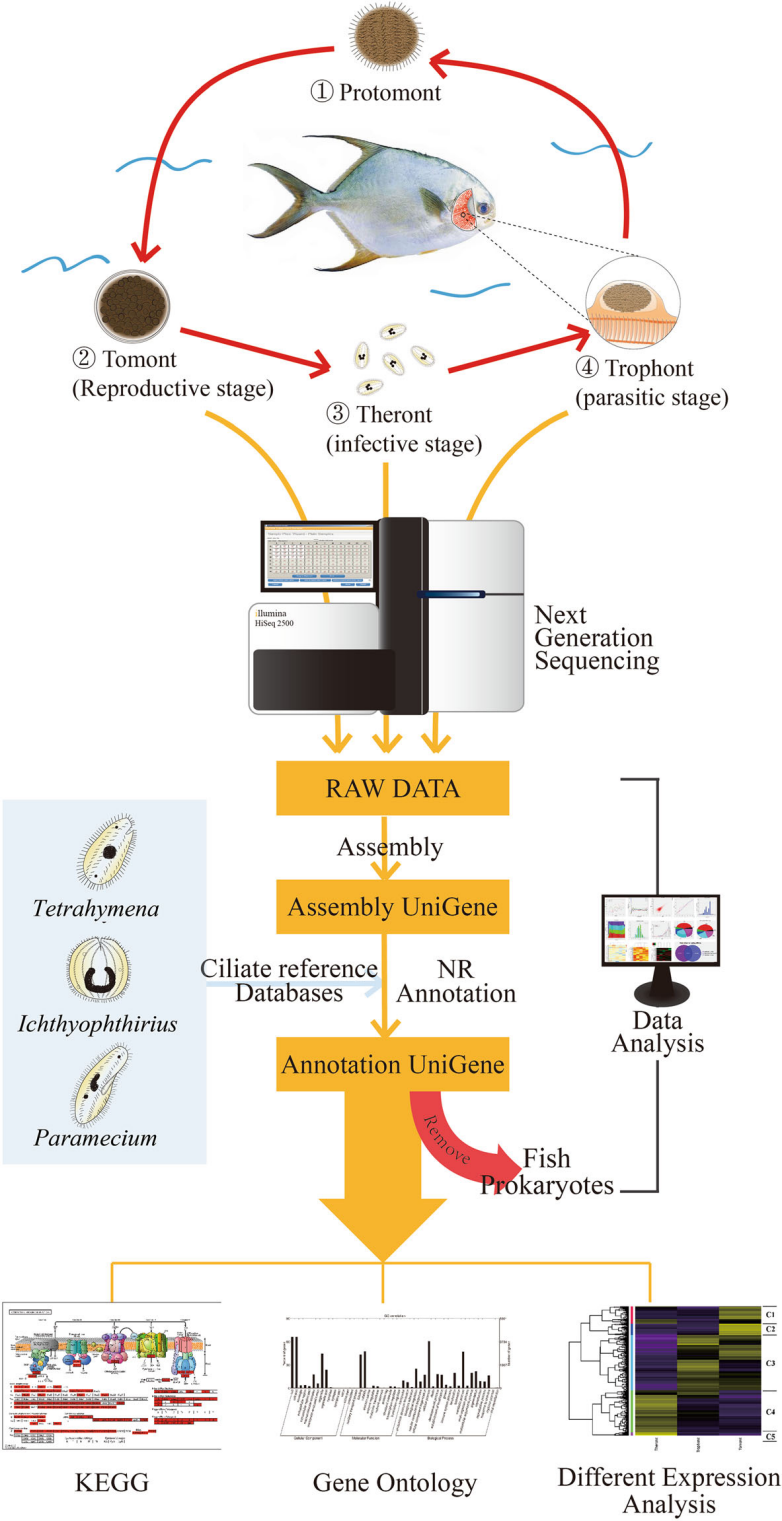
Life-cycle of *C. irritans* and RNA-Seq. Infective theront-stage parasites in seawater invade the gills and skin of host fish then rapidly develop into parasitic trophonts within the host epidermis. After 2–3 days of parasitic feeding, mature trophonts exit the infection site and grow into protomonts, which attach to an inert support, then rapidly transform into reproductive tomonts and divide into new infective theronts within 48–60 h at 27 °C. Sequences identified in the theront, trophont, and tomont stages were subjected to gene annotation, GO function annotation, KEGG analysis, DEG and cluster analysis

The protozoan *Cryptocaryon irritans* is one of the most common parasitic ciliates. It can infect many species of marine fish in tropical and subtropical regions, causing cryptocaryonosis [1–4]. Although the parasite does not usually cause severe infections in wild fish, it has become a major problem in aquaria and aquaculture industries. In South China, direct economic losses due to cryptocaryoniasis have amounted to over 16 million US dollars just in the Guangdong Province each year (data not published). *Cryptocaryon irritans* has four main life-cycle stages (Fig. 1): parasitic trophont, off-host protomont, reproductive tomont and infective theront [3]. The trophont lives within the host epithelium, and the typical clinical signs of cryptocaryonosis, including pinhead-sized white nodules covering the surface of the skin, gills and eyes, can be observed by the naked eye during this stage. The mature trophont leaves the host and becomes a protomont before encysting and transforming into a tomont, which then attaches to the substrate, develops, and divides into numerous daughter tomites by asymmetric binary fission. These tomites subsequently leave the cyst as theronts, which actively seek new fish hosts and burrow into the epithelium to start the next life-cycle. An entire life-cycle takes one week in *Trachinotus ovatus* hosts used to maintain *C. irritans* at 27 °C in artificial conditions [5].

Despite major losses caused by cryptocaryonosis, there is currently no effective means of controlling this disease [6]. However, previous studies indicated that *C. irritans* can regulate the expression of host immune-related genes [7–12], and immunization with *C. irritans* can confer protection [13–15], implicating immune prophylaxis as a potential alternative method of control. However, it is impractical to culture *C. irritans* continuously in vitro in large-scale industrial operations, and using fish as hosts to passage the parasite is both time-consuming and costly. Surface proteins called immobilization antigens (I-antigens) were recently identified in this parasite, and a modified DNA vaccine based on these antigens was demonstrated to protect fish against infection by *C. irritans* [16]. However, no other potential antigen proteins have yet been identified.

Information on the transcriptome of a parasite can help to discover genes and understand the molecular processes involved in parasite development, reproduction and host interactions, as well as facilitating the search for potential vaccine candidates and drug targets [17–22]. Comparative analysis of *Ichthyophthirius multifiliis* trophonts and theronts showed that the most abundant transcripts were highly stage-specific and coincided with metabolic activities [21]. In 2010, Lokanathan et al. [22] identified 2,659 expressed sequence tags (ESTs) from a cDNA library of *C. irritans* tomonts, of which just 1,611 matched proteins with known functions. Although these results offered insight into the genomics of *C. irritans*, they only identified gene sequences, with little information about the transcript levels of each gene, or the genes involved in different stages of the life-cycle. The present study therefore aimed to obtain a more complete transcript picture of *C. irritans* by sequencing mRNA from all three life-cycle stages (theront, trophont, and tomont) using the Illumina RNA-seq method. A total of 9,253 high-quality unigenes were identified that significantly matched other ciliate proteins. We analyzed the expression profiles of these unigenes and described their differential expression patterns in the three stages. The results of this study improve our understanding of *C. irritans* biology and will help to further the development of effective methods for controlling cryptocaryonosis.

## Methods

### Parasite preparation

*Cryptocaryon irritans* for sequencing were originally isolated from an infected *T. ovatus* obtained from a local farm in Daya Bay, Guangdong Province, China, and maintained by serial passage using *T. ovatus* as hosts, as described previously [5]. To collect parasites at different life stages, mature trophonts were gently scraped from infected *T. ovatus* gills (3 days post-infection) and carefully washed to remove contaminating tissue debris. The trophonts were then incubated in sterilized seawater at 28 °C for 12 h to develop into tomonts, or for 3 days to develop into theronts. After centrifugation at 800× *g* for 5 min, trophonts and tomonts were collected. Theronts were cooled in an ice-bath for 30 min, and then harvested after centrifugation at 4,000× *g* for 5 min. All *C. irritans* were immediately cryopreserved in liquid nitrogen until RNA isolation.

### RNA extraction

RNA was extracted from trophonts, tomonts, and theronts using an RNeasy Mini Kit (Qiagen, Hilden, Germany) according to the manufacturer’s instructions. The quantity and integrity of the RNA were determined using a NanoDropND-1000 spectrophotometer (Thermo, Waltham, USA) and Agilent 2100 Bioanalyzer (Agilent Technologies, Richardson, USA), respectively, and RNAs with a RNA Integrity Number (RIN) > 8, 28S/18S > 0.7, and A260/280 values of about 2.0 were used to prepare the libraries.

### Library construction and sequencing

Sequencing libraries were constructed according to the TruSeq® RNA Sample Preparation v2 manuals (Illumina, San Diego, USA). Briefly, poly(A)^+^ mRNA was purified from the total RNA with oligo(dT) attached magnetic beads, and cleaved into small fragments. First-strand cDNA was synthesized using the fragmented RNA as template and random hexamers as primers, and second-strand cDNA was obtained using Second Strand Master Mix (Thermo, Waltham, USA). After repairing the overhang ends into blunt ends with End Repair Mix, a single “A” nucleotide was added to the 3′ end of the double-stranded cDNA, followed by ligation to an adapter containing a single “T”. Polymerase chain reaction (PCR) was used to enrich the DNA fragments with adapter molecules on both ends and amplify the amount of DNA in the library. The concentrations and sizes of the libraries were measured with a Qubit®2.0 Fluorometer (Life Technologies, Camarillo, USA) and Agilent 2100 Bioanalyzer, respectively. The libraries were then sequenced and analyzed using an Illumina HiSeq™ 2500 instrument (Illumina, San Diego, USA) with paired end 2 × 100 nucleotide multiplex, according to the manufacturer’s instructions [23] (Fig. 1).

### *De novo* assembly and gene annotation

Adaptors, low-quality reads (> 50% bases with quality (Q) value ≤ 10), ambiguous nucleotide reads (> 10% ‘N’ rate, where ‘N’ represents ambiguous bases in reads), and sequence reads < 20 bp were removed. All clean reads from the three stages served as a pool reads and were processed with CLC Genomics Workbench software [24–26] using the scaffolding contig program (word-size = 45, minimum contig length ≥ 300) and CAP3 EST software to generate *de novo* assembled unigenes. All unigenes were first compared with the *T. ovatus* transcriptome database (http://www.ncbi.nlm.nih.gov/sra/SRX534464) to remove host-cell contamination. A bacterial database (total 222,262,566 proteins) was then downloaded from a non-redundant (NR) protein database to remove prokaryote contamination, with a cut-off E-value for contaminating contigs of < 1e^-5^ and identity ≥ 70% in the above analysis. Finally, the clean unigenes were annotated using a high-throughput BLASTx program against the NR protein database, and protein databases of *Tetrahymena thermophila* (http://www.ciliate.org/system/downloads/T_thermophila_June2014_proteins.fasta), *Tetrahymena borealis* (http://www.ciliate.org/system/downloads/T_borealis_oct2012_proteins.fasta), *Tetrahymena elliotti* (http://www.ciliate.org/system/downloads/T_elliotti_oct2012_proteins.fasta), *Tetrahymena malaccensis* (http://www.ciliate.org/system/downloads/T_malaccensis_oct2012_proteins.fasta), *Paramecium tetraurelia* (http://paramecium.cgm.cnrs-gif.fr/download/fasta/Ptetraurelia_peptides_cur.fasta), and *I. multifiliis* (http://ich.ciliate.org/system/downloads/img1_0407.aa.fsa) (E-value < 1e^-5^, identity ≥ 30% and coverage ≥ 50%). Potential C-terminal glycosylphosphatidylinositol (GPI)-modification sites were predicted using big-II predictor (http://mendel.imp.ac.at/sat/gpi/gpi_server.html) [27]. Gene ontology (GO) annotation was performed using Blast2GO and GO enrichment analysis using top GO package. Kyoto Encyclopedia of Genes and Genomes (KEGG) pathways were obtained from KEGG databases.

### DEGs and cluster analysis

The relative expression levels of unigenes were calculated as reads per kilobases per million reads (RPKM) [28]. To compare the pronounced active transcripts among the three stages of *C. irritans*, the *edegR* package was used to define significantly upregulated or downregulated genes with a threshold value of fold change ≥ 4 and false discovery rate (FDR) < 0.001 [29]. Based on the expression pattern, cluster analysis of differentially expressed genes (DEGs) in the three stages was performed using *Cluster* package. A heat map was generated using R, and a GO annotation plot was generated using WEGO (http://wego.genomics.org.cn/cgi-bin/wego/index.pl) [30].

### Experimental validation of transcription levels

To confirm the RNA-seq results, gene expression levels were determined using the LightCycler480 real-time PCR system (Roche, Mannheim, Germany) using SYBR Green Realtime PCR Master Mix (Thermo, Waltham, USA), according to the manufacturer’s instructions, as described previously [31]. Total RNA was extracted from each parasite stage using an RNeasy Mini Kit, as described above, and stored at -80 °C. cDNA was synthesized from total RNA for each parasite stage using a ReverTra Ace-α-Kit (Toyobo, Osaka, Japan), according to the manufacturer’s protocol. Eight gene-specific primers were designed based on the transcriptome library (Additional file 1: Table S1). Elongation factor-1β (EF-1β) primers (EF-1β RTF/EF-1β RTR) were introduced as the reference gene. The cycling protocol was as follows: 94 °C for 2 min, and 94 °C for 15 s, 58 °C for 15 s, and 72 °C for 20 s for 40 cycles. Melting-curve analysis was used to detect the specificity of the PCR products, which was verified by sequencing. All samples were analyzed in triplicate. The expression of each target gene was normalized to the housekeeping gene EF-1β, calculated according to the 2^-ΔΔCt^ method [32]. Tomont samples were used as the calibration control. Thus, ΔΔCt = [(Ct_target_ – Ct_EF-1β_)]_trophont or theront stage_ – [(Ct_target_ – Ct_EF-1β_)] _tomont stage_. All data were analyzed using SPSS (version 16.0) software. Correlations between quantitative PCR (qPCR) and RNA-seq results were determined using Pearson’s *r*.

## Results

### Sequencing and de novo assembly

To examine the transcriptional profile of *C. irritans*, we constructed three cDNA libraries using total poly(A)^+^ mRNA isolated from trophonts, tomonts, and theronts, respectively. Totals of 79.35, 66.42, and 123.62 million raw reads, respectively, were produced by Illumina Hiseq 2500 sequencing (Table 1). All raw reads were submitted to the Sequence Read Archive database at NCBI (SUB1416064, SUB1416075, and SUB1416142). After removal of adaptors, low-quality reads, ambiguous reads, and reads < 20 bp, 73.15, 62.23, and 109.57 million clean reads were generated from the trophont, tomont, and theront libraries, respectively, with a clean-read ratio of 90.92%. A total of 49,104 unigenes were then obtained after *de novo* assembly, with a total length of 50,174,026 bp, an average length of 1,022 bp, and N50 of 1,338 bp.

**Table 1:** Summary statistics of *C. irritans* transcriptome

	Tomont	Theront	Trophont
Sequencing			
Raw reads (pair-end)	79,350,336	66,432,722	123,624,738
Clean reads (pair-end)	73,151,792	62,228,442	109,567,740
Clean ratio	92.19%	93.67%	88.63%
Assembly			
	Primary UniGene		Final UniGene
No. of unigenes	80,078		49,104
Total length (bp)	76,079,839		50,174,026
N50 length (bp)	1,302		1,338
Mean length (bp)	1,002		1,022
Largest transcripts (bp)	17,074		17,074
Eliminating contamination			
	No. of clean unigenes		
Not *T. ovatus*	31,893		
Not prokaryote CDS (≥ 100 aa)^a^	9,253		
Annotation			
NCBI-Nr	8,569		
	No. of best hits among 6 ciliate reference databases		Total no. of the reference databases
*I. multifiliis*	1,551		8,097
*P. peptides*	2,036		39,519
*T. borealis*	1,867		21,943
*T. elliotti*	1,332		22,562
*T. malaccensis*	1,252		26,378
*T. thermophila*	1,215		26,996

^a^CDS (≥ 100 aa): only used the CDS region more than 300 bp

### Unigene annotation

**Fig. 2:**
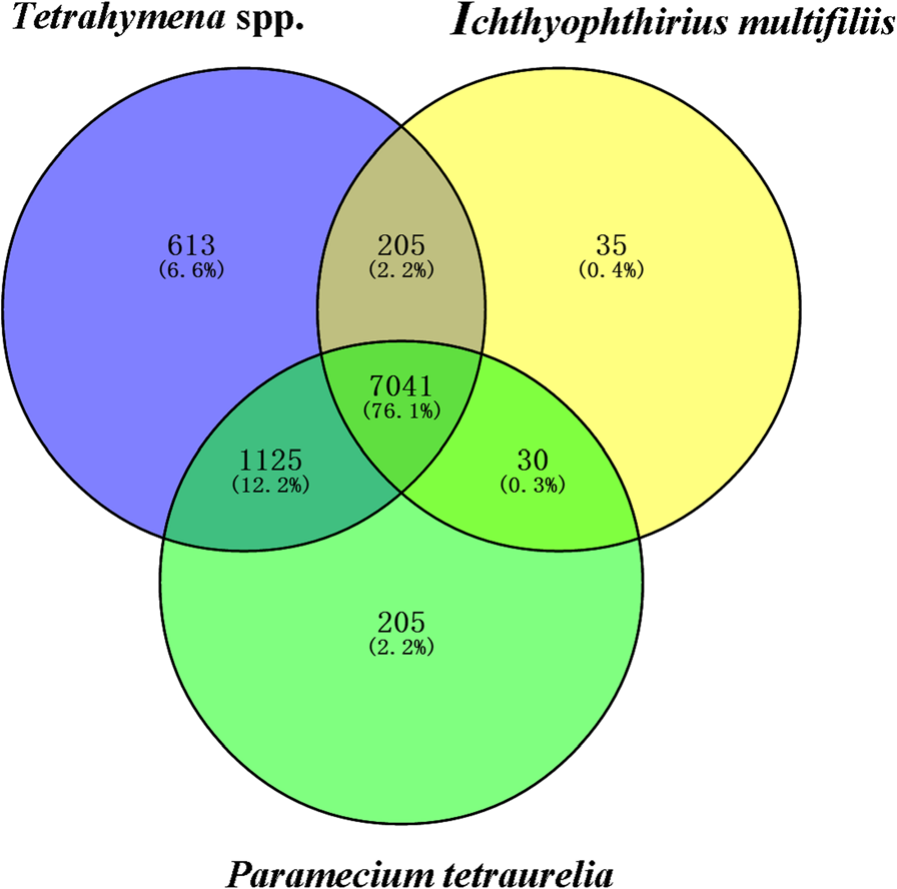
*Cryptocaryon irritans* proteins shared among *I. multifiliis*, *P. tetraurelia* and *Tetrahymena* spp. (four species in total). Venn diagram summary of *C. irritans* unigenes in comparison with six ciliate peptide databases. Numbers in the overlapping areas represent matching peptides (E-value < 1e^-5^) in the relevant organisms with the query 9,253 unigenes of *C. irritans*

To obtain more *C. irritans*-derived genes, the unigenes were first compared with a *T. ovatus* transcriptome database to obtain 31,893 non-host contigs, followed by a bacterial database to remove the prokaryote contamination. This left 9,253 contigs (Coding sequence ≥ 100 amino acids) (Table 1), of which 8,569 contigs matched entries in the NR protein database, and all 9,253 contigs (Additional file 1: Tables S2 and S3) matched to the six ciliate databases, corresponding to the number of *I. multifiliis* annotations [33, 34]. A Venn diagram summarizing *C. irritans* unigenes compared with peptide sequences from *Tetrahymena* (four species in total), *I. multifiliis*, and *P. tetraurelia* is shown in Fig. 2. A total of 7,041 (76.1%) unigenes were shared by all ciliate databases.

### GO function annotation

**Fig. 3:**
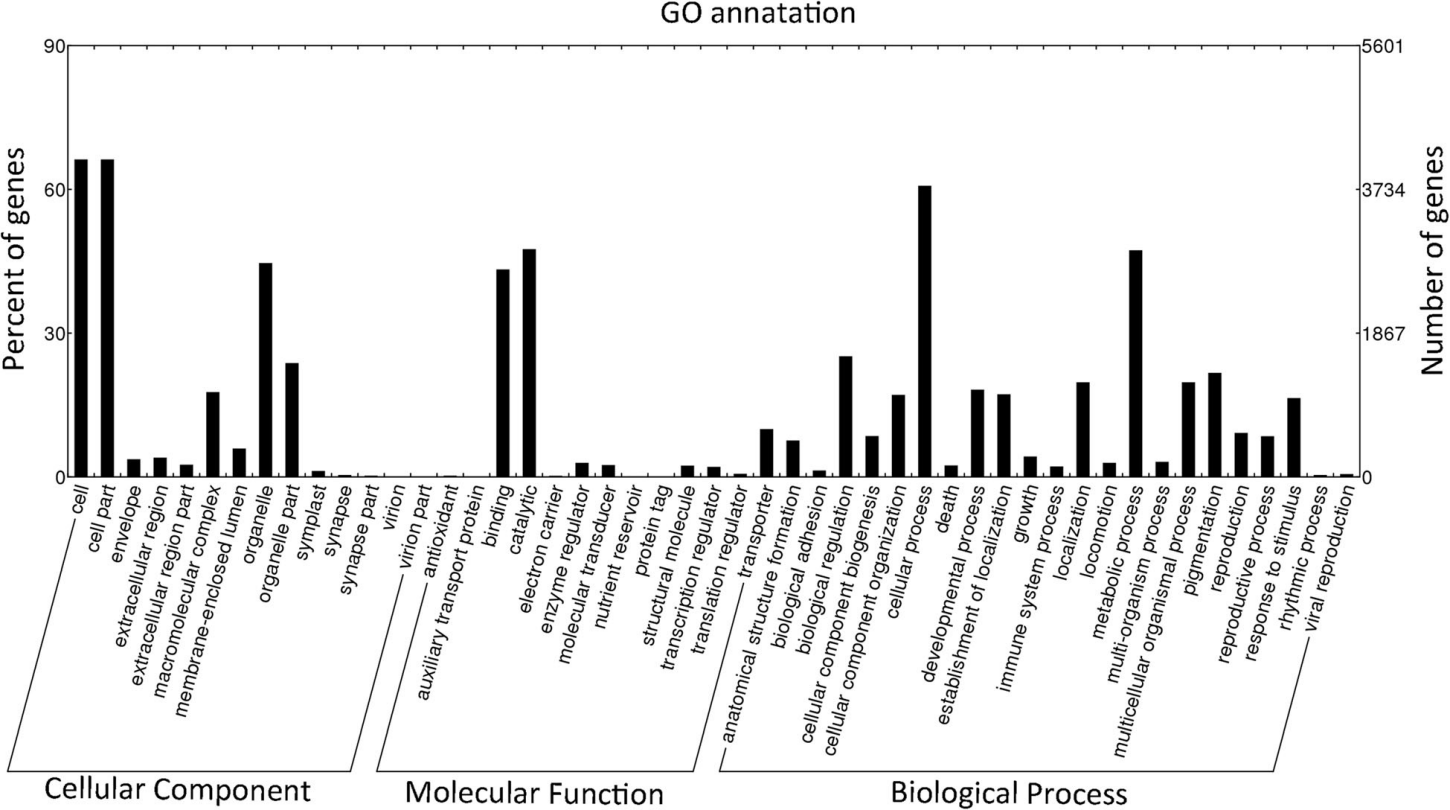
Gene ontology (GO) annotation. Distribution of level 2 GO terms including biological process, molecular function and cellular component among all annotated genes

For functional predictions and categories, all 9,253 unigenes were annotated with 43,436 GO terms and assigned into three functional GO terms, including cellular component (14 sub-categories), molecular function (13 sub-categories), and biological process categories (22 sub-categories) (Fig. 3 and Additional file 1: Table S4). Cell part (66.3%) was the largest sub-category in the cellular component category, while the main sub-categories in molecular function were catalytic activity (47.6%) and binding (43.3%), and the major biological processes were cellular process (60.7%) and metabolic process (47.3%).

### KEGG enrichment analysis

Mapping the reference pathways in the KEGG database identified a total of 230 KEGG pathways (Additional file 1: Table S5). The pathways with most genes were the ribosome, spliceosome, carbon metabolism, and purine metabolism pathways, which was in accord with the previous transcriptome analysis of *C. irritans* tomonts [35]. Comparisons with the *T. ovatus* transcriptome [36] and other ciliate genomes indicated that the overall metabolism of ciliates was largely identical, with only minor differences.

### DEGs and cluster analysis

**Fig. 4:**
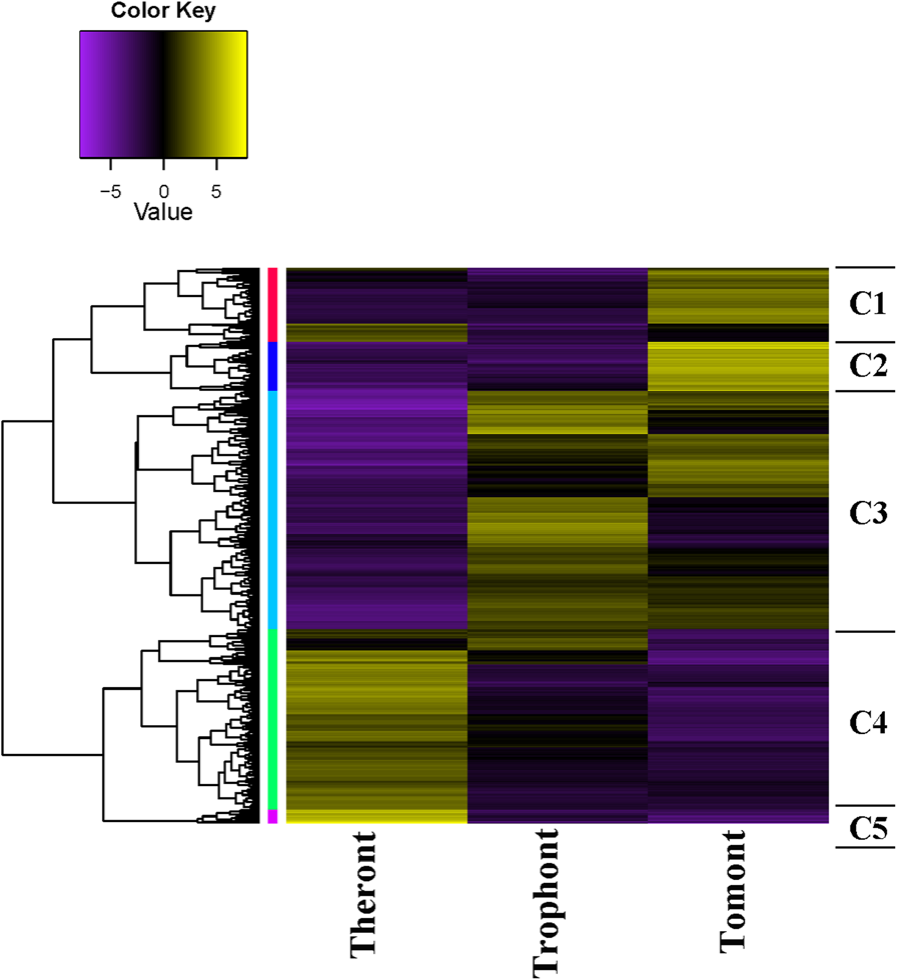
Heat map of DEG clusters

**Fig. 5:**
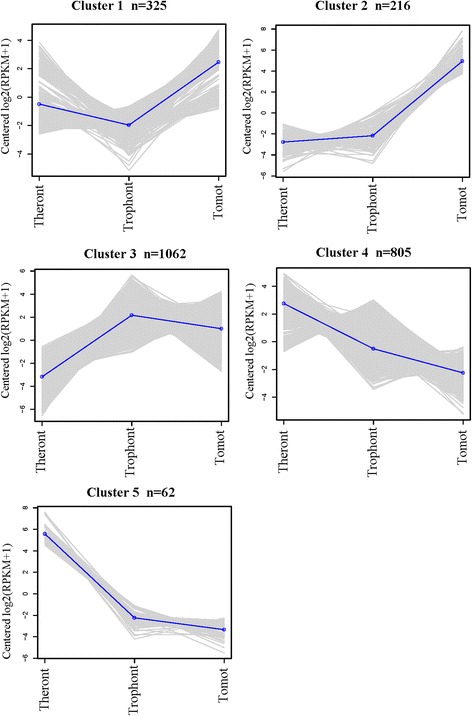
Number of DEGs and their expression patterns in each cluster. Based on the expression patterns, cluster analysis of DEGs in each life-cycle stage was determined using Cluster package

In the present study, we used a threshold of FDR < 0.001 and absolute fold change ≥ 4 to define significantly upregulated or downregulated genes in pairwise comparisons. A total of 2,470 DEGs were identified across the three stages, including 2,011 genes that were significantly differentially expressed between tomonts and theronts, comprising 1,103 upregulated and 908 downregulated genes. A total of 1,404 DEGs, including 631 upregulated and 773 downregulated genes in the tomont/trophont pairwise comparison, and 1,797 DEGs, including 805 upregulated and 992 downregulated genes in the theront/trophont pairwise comparison. Based on hierarchical clustering results, we generated a heat map to illustrate the differential gene expression patterns in the three stages (Fig. 4). DEGs formed five major clusters (Fig. 5 and Additional file 1: Table S6). DEGs in clusters 1 and 2 were more highly expressed in tomonts than in the other stages, while DEGs in cluster 3 had lower expression levels in theronts but relatively higher expression levels in tomonts and trophonts, while clusters 4 and 5 showed increased expression in the theront stage.

**Fig. 6:**
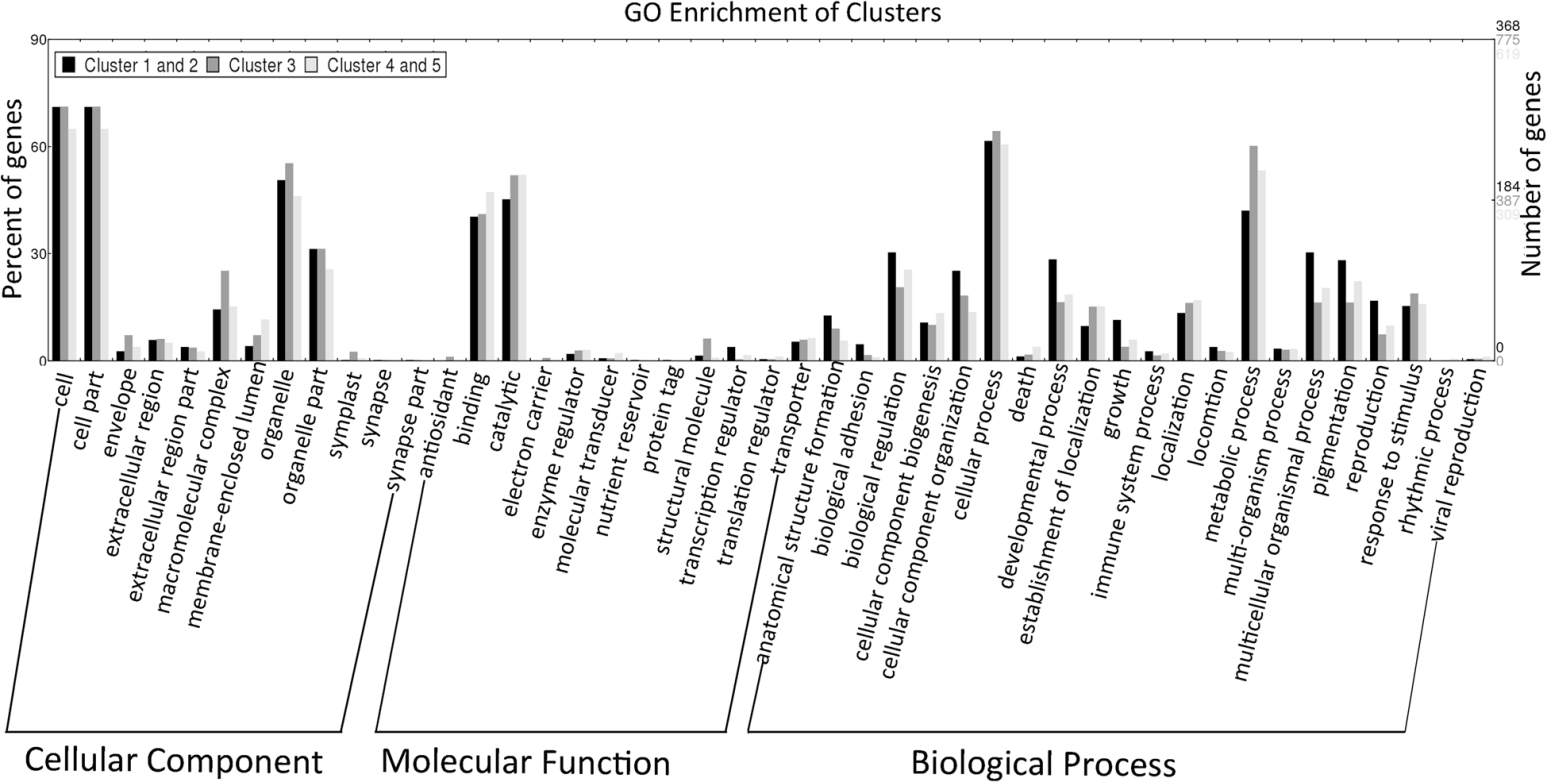
Comparative analysis of GO annotation level 2 terms associated with clusters 1 and 2, cluster 3, and clusters 4 and 5, respectively. DEGs in clusters 1 and 2 were associated with developmental process and reproduction; DEGs in cluster 3 were associated with response to stimuli and localization; and DEGs in clusters 4 and 5 were also enriched in response to stimuli

GO enrichment analysis (Fig. 6) revealed that DEGs in clusters 1 and 2, including the genes encoding histone H2A variant 3, protein kinase 2, Cyclic Adenosine monophosphate (cAMP)-dependent protein kinase regulatory subunit, cat-eye syndrome critical region protein 2, translationally-controlled tumor protein homolog, calpain-type cysteine protease DEK1, and calpain-type cysteine protease ADL1 were enriched in biological processes of reproduction and regulation of cell growth. DEGs such as those encoding phosphatidylinositol-4-phosphate 5-kinase 1, phosphatidylinositol-4-phosphate 5-kinase 4, phosphatidylinositol-4-phosphate 5-kinase 6, phosphatidylinositol-4-phosphate 5-kinase 9, and SNF1-related protein kinase catalytic subunit alpha KIN10, which were involved in lipid metabolism, were also enriched in those clusters.

DEGs in cluster 3, such as those involved in glycometabolism (glycogen phosphorylase 1, glycogen debranching enzyme, phosphoglucomutase-2, glucose-6-phosphate isomerase, cytosolic 1), lipid metabolism (acetyl-CoA acetyltransferase, hormone-sensitive lipase, phosphoglycerate kinase), amino acid metabolism (aspartate aminotransferase, alanine aminotransferase, glutamine-fructose-6-phosphate aminotransferase) were largely enriched in the metabolism of nutrient substances. In addition, genes such those encoding 40S ribosomal protein S3-3, aconitate hydratase 2, aldehyde dehydrogenase family 7 member B4, endoplasmin homolog, proteasome subunit beta type-4, shaggy-related protein kinase epsilon, and sodium/hydrogen exchanger 7, which were involved in the biological process of response to abiotic stimulus, salt stress and osmotic stress, were also increased in cluster 3.

DEGs in clusters 4 and 5, including those for calpain-D, E3 ubiquitin-protein ligase CHIP, SNW/SKI-interacting protein, cation/calcium exchanger 3, mitogen-activated protein kinase 2 (MAPK2), MAPK 3, MAPK 4 and serine/threonine-protein kinase AtPK1/AtPK6 were enriched in response to osmotic stress, abiotic stimuli, or salt stress. Genes for aspartic proteinase-like protein 1, aspartic proteinase-like protein 2, serine carboxypeptidase-like 47, serine carboxypeptidase-like 49, serine/threonine-protein kinase AFC1, ubiquitin carboxyl-terminal hydrolase 4 and methionine aminopeptidase 2B, which were involved in proteolysis and protein modification process, were also increased in cluster 4.

### Identification of I-antigens and proteases

I-antigens and proteases are recognized as major targets for vaccine development and potential drug targets in parasites, respectively. A total of nine putative I-antigens were found in our transcriptome (Additional file 1: Table S3). Two I-antigen transcripts were highly expressed in all three stages of *C. irritans* (RPKM > 200), and shared only 57% similarity with each other. In addition, 122 proteases were found in the transcriptome, most of which were calpain family cysteine proteases (82). Six cysteine proteases were highly expressed (RPKM > 100) in trophonts.

### qPCR validation

**Fig. 7:**
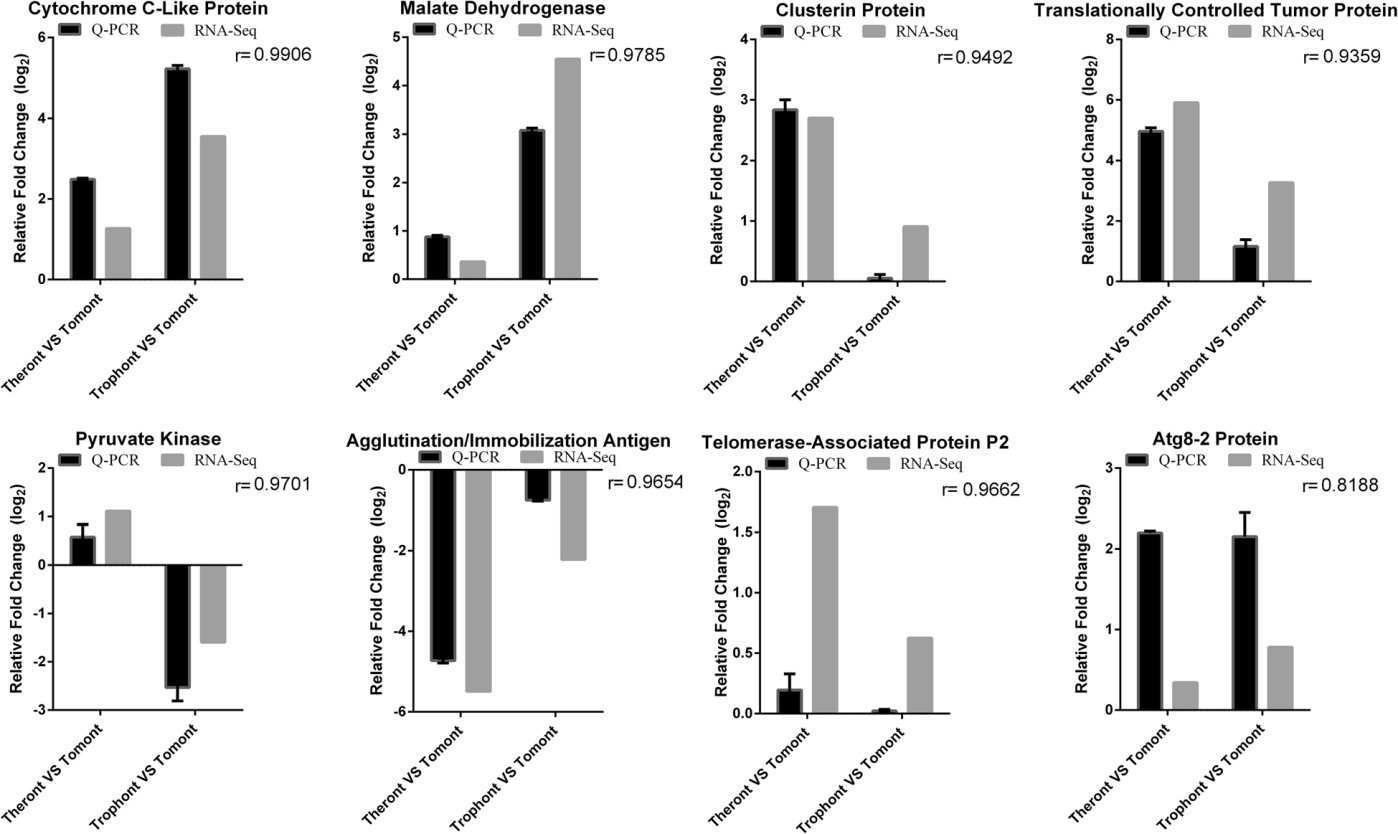
Expression profiles of eight genes in different life-cycle stages from RNA-Seq (black) and qPCR (gray), with EF-1β as reference gene

Eight genes were randomly selected from the transcriptome library to verify the transcription levels in each stage by qPCR. After normalizing to the EF-1β gene, the relative expression levels were presented as the fold-change relative to the tomont stage. The correlation coefficient (*r*) between RNA-Seq and qPCR ranged from 0.8188 to 0.9906, depending on the specific gene and reference gene (Fig. 7).

## Discussion

Understanding the comparative transcriptional profile of *C. irritans* is an essential goal for parasite physiology and the prevention of cryptocaryonosis. In this study, we performed comparative gene transcription analysis among the three life-cycle stages of *C. irritans*. To obtain a more complete reference transcriptome, all raw reads from the three stages were first pooled and *de novo* assembled. A more specific *C. irritans*-derived transcriptome was obtained by removing contamination from host fish and prokaryotes by comparison with a *T. ovatus* transcriptome database and a bacterial database. A total of 9,253 unigenes were obtained, 7,041 of which were shared by other ciliate databases. A comprehensive repertoire of gene annotation, GO function annotation, KEGG analysis, DEG, and cluster analysis was conducted.

The macronuclear genome of *I. multifiliis* was published in 2011 [33] and is available as a reference database for *I. multifiliis* mapping. However, there is currently no available *C. irritans* reference database for mapping, and we therefore used the *de novo* method to assemble the *C. irritans* transcriptome. Moreover, *C. irritans* must be cultured in host fish, and we recently demonstrated that nonspecific cytotoxic cells receptor protein^+^ (NCCRP^+^) cells can be found inside food vacuoles in trophonts [35]. In addition, *C. irritans* cannot be cultured axenically, and contamination by host RNA and/or RNA from endosymbiotic bacterium cannot be completely eliminated when preparing samples. Although oligo (dT) magnetic beads were used to enrich the eukaryotic mRNA, almost half the assembled unigenes still matched to prokaryotic genes (22,640). A similar study in *I. multifiliis* showed that 17.2% of ESTs in the *I. multifiliis* transcriptome strongly matched the genome sequence of an endosymbiotic bacterium [34, 37]. A previous report of *C. irritans* transcriptome data also showed that only 57% of unique transcripts matched to other ciliate species [35]. We cannot fully explain the reason for the high proportion of prokaryote transcripts in our transcriptome, but focused on those unigenes that significantly matched to other ciliate species.

Tomonts represent a crucial stage in the life-cycle of *C. irritans*, ensuring continuity of the parasite. In this stage, asynchronous theronts are released from tomonts post-encystment, and the maximum yield of theronts was 122-fold in in vitro propagation under experimental conditions [38]. DEGs in clusters 1 and 2 were more highly expressed in tomonts. Consistent with a previous report in *C. irritans* [35], GO enrichment analysis showed that DEGs in clusters 1 and 2 were enriched in the biological processes of reproduction and regulation of cell growth, which are the major biological activities in cells during the tomont stage. Some genes involved in lipid metabolism were also enriched in clusters 1 and 2. Given that tomonts cannot obtain energy substrates from the external environment, the role of lipids as the major energy substrate in tomonts needs further investigation.

Trophonts are the only parasitic stage during the life-cycle of *C. irritans*, and need to acquire enough energy for the forthcoming reproductive tomont stage. DEGs in cluster 3 were highly expressed in the trophont and tomont stages, and were largely enriched in the utilization of nutrient substances. For protozoan parasites, the parasitic stage is an important state for acquiring nutrition from their host, and genes involved in metabolic processes thus dominate cellular activity. A previous transcriptional analysis of the model protozoan *T. thermophila* demonstrated that 60 of 148 selected representative genes were upregulated in the growth stage and were involved in metabolic processes [23]. Approximately 30% of sequences in the parasite *Trypanosoma vivax* showed significantly high expression levels in the bloodstream stage, and were confirmed to be genes related to metabolic processes [39]. Transcriptomic analysis of *I. multifiliis* revealed that transcripts related to metabolic enzymes, including succinyl-CoA ligase, aldo/keto reductase, and glutamine synthetase, were significantly upregulated in the trophont stage [21]. In the present study, those metabolic enzymes were also highly expressed in the trophont stage. Highly expressed unigenes (RPKM > 100, 352 unigenes) encoding metabolic enzymes may relate to the importance of energy metabolism and material exchange in the cellular activity of parasitic trophonts. The oral route is a convenient way to apply medicines, and given that trophonts are the only parasitic stage during the life-cycle of *C. irritans*, they represent the ideal stage at which to administer oral drugs [40, 41]. Inhibitors of the proteins involved in trophont metabolic pathways may thus provide a means of controlling this parasite infection. In addition, DEGs involved in the biological processes of response to abiotic stimuli, salt stress, and osmotic stress were also increased in cluster 3, indicating that those genes may relate to the adjustment of permeation pressure during the transition from host body to saltwater, when the trophont leaves the host fish and develops into a tomont.

Among the three life-cycle stages of *C. irritans*, theronts are responsible for host invasion. DEGs in clusters 4 and 5 were more highly expressed in the theront stage. DEGs in cluster 4 were enriched in response to osmotic stress, abiotic stimuli, and salt stress. The active transcription of these genes in cluster 4 may reflect a series of stress reactions in response to exposure to saltwater, and may be involved in acclimating theronts to the change in permeation pressure during the transition from tomont to saltwater. Notably, we cooled the theronts in an ice-bath for 30 min for sample preparation, which may have affected transcription. However, genes related to response to temperature stimuli did not appear to be enriched in theronts. Protein metabolism was also active in cluster 4, and DEGs involved in proteolysis and protein modification were more highly transcribed in this cluster, suggesting that efficient protein metabolism is crucial for theronts exposed to saltwater. In addition, transcripts such as those for patatin family phospholipases and mitochondrial carrier proteins, which are reportedly involved in the balance of energy usage/storage [42, 43], were highly transcribed in clusters 4 and 5. Given that theronts cannot survive for long without finding a host, it seems that theronts require a careful energy balance during host finding.

Along with the DEGs described above, some transcripts highly expressed in all three stages of *C. irritans* merit additional consideration. I-antigens are well-studied surface proteins in some ciliates. Although their functions remain unknown, I-antigens in *I. multifiliis* have been shown to elicit protective immunity in fish and have long been considered major targets for vaccine development [44, 45]. *Cryptocaryon irritans* has previously been shown to elicit effective host systematic and skin humoral immune responses [46–48], and some putative I-antigens have been identified in *C. irritans* [49–51]. Vaccination with DNA or recombinant I-antigen vaccine improved post-challenge survival by 46% [16]. In this study, we found nine putative I-antigen transcripts in our *C. irritans* transcriptome, which were expressed at various levels in the three stages (Additional file 1: Table S3). Two I-antigen transcripts were highly expressed in all three stages of *C. irritans* (RPKM > 200), and shared only 57% similarity with each other, though a potential C-terminal GPI-modification site was predicted in both transcripts. Further studies are needed to determine if these proteins share similar functions. These newly identified I-antigens provide more potential targets for vaccine development against *C. irritans*.

Proteases play crucial roles in parasite infection and development. Because of the feasibility of designing specific inhibitors, proteases have long been recognized as potential drug targets in parasites [33, 52–54]. For example, the synthetic peptide GlcA-Val-Leu-Gly-Lys-NHC_2_H_5_ effectively inhibited *Plasmodium falciparum* schizont cysteine protease Pf 68 in vitro [52]. α2-Macroglobulin (α2M), a non-specific protease inhibitor of endogenous and exogenous proteases, was found to be involved in the immune response to *I. multifiliis* in infected carp [53]. We previously established a transcriptome database of skin, gill, spleen and head kidney for the grouper *Epinephelus coioides* at different time points after *C. irritans* infection [54, 31]. We found three α2M isoforms in groupers, all of which were significantly upregulated in grouper gill and spleen post-infection (data unpublished). However, the role of α2M in the defense against *C. irritans* infection remains to be demonstrated. Nevertheless, these results suggest that anti-protease drugs could be exploited to control this parasite. We identified 122 protease transcripts in our transcriptome, most of which (82 transcripts) were calpain family cysteine proteases, thus significantly expanding the range of potential therapeutic targets for protease inhibitors. Calpain family cysteine proteases appear to play important roles in *C. irritans*, suggesting that they should be considered as the primary protease inhibitor targets. In addition, six proteases were highly expressed (RPKM > 100) in trophonts, and only shared about 30% amino acid similarity with teleosts. This suggests that specific inhibitors against *C. irritans* proteases could be designed based on those unigenes.

## Conclusions

We established a reference transcriptome of *C. irritans* and characterized the genes and their functional categories at each stage of the parasite’s life-cycle. A total of 9,253 unigenes were generated by *de novo* assembly followed by removal of contamination and multiple annotations. A total of 2,470 DEGs were identified across the three life-cycle stages. DEGs enriched in tomonts were associated with cell division, while DEGs enriched in theronts were involved in response to stimuli, and DEGs enriched in trophonts were related to the accumulation of nutrients and cell growth. In addition, the identification of proteases and putative I-antigen transcripts in the transcriptome could contribute to vaccine development or drug targeting. Further studies are planned focusing on functional verification of the important DEGs in each life-cycle stage of *C. irritans*, with a view to developing effective vaccines or drugs to control this disease.

## Additional file

Additional file 1: Table S1. Primers used in this study. **Table S2.** Gene annotation using ciliate databases. **Table S3.** Gene annotation using NR database. **Table S4.** GO function annotation. **Table S5.** KEGG annotation. **Table S6.** Number of DEGs in clusters. (XLSX 2815 kb)

## Abbreviations

α2M: Alpha-2-macroglobulin
DEG: Differentially expressed gene
EF-1β: Elongation factor-1 β
FDR: False discovery rate
GPI: Glycosylphosphatidylinositol
I-antigens: Immobilization antigens
MAPK: Mitogen-activated protein kinase
RPKM: Reads Per Kilo bases per Million reads
